# Anionic liposome formulation for oral delivery of thuricin CD, a potential antimicrobial peptide therapeutic

**DOI:** 10.1016/j.ijpharm.2024.123918

**Published:** 2024-04

**Authors:** Camila Viera Herrera, Paula M. O’Connor, Poonam Ratrey, R. Paul Ross, Colin Hill, Sarah P. Hudson

**Affiliations:** a Department of Chemical Sciences, Bernal Institute, University of Limerick, Ireland; b Food Biosciences, Teagasc, Moorepark Food Research Centre, Fermoy, Co. Cork, Ireland; c APC Microbiome Ireland Cork, Cork, Ireland; d School of Microbiology, University College Cork, College Road, Cork, Ireland

**Keywords:** Thuricin CD, Antimicrobial peptides, Bacteriocins, Antimicrobial resistance, Liposomes

## Abstract

Thuricin CD is a two-peptide antimicrobial produced by *Bacillus thuringiensis*. Unlike previous antibiotics, it has shown narrow spectrum activity against *Clostridioides difficile*, a bacterium capable of causing infectious disease in the colon. However, peptide antibiotics have stability, solubility, and permeability problems that can affect their performance *in vivo*. This work focuses on the bioactivity and bioavailability of thuricin CD with a view to developing a formulation for delivery of active thuricin CD peptides through the gastrointestinal tract (GIT) for local delivery in the colon. The results indicate that thuricin CD is active at low concentrations only when both peptides are present. While thuricin CD was degraded by proteases and was unstable and poorly soluble in gastric fluid, it showed increased solubility in intestinal fluid, probably due to micelle encapsulation. Based on this, thuricin CD was encapsulated in anionic liposomes, which showed increased activity compared to the free peptide, maintained activity after exposure to pepsin in gastric fluid and intestinal fluid, was stable in suspension for over 21 days at room temperature and for 60 days at 4 °C, and exhibited no toxicity to epithelial intestinal cells. These findings suggest that an anionic lipid-based nano formulation may be a promising approach for local oral delivery of thuricin CD.

## 1 Introduction

Overprescription of antibiotics, along with their overuse in livestock agriculture as growth promoters, combined with very few antimicrobial therapies in the drug development pipeline, has contributed to the development of resistance mechanisms in bacteria (Ventola, 2015). If left unaddressed, drug-resistant infections are expected to contribute to the mortality of approximately 10 million people globally per annum by 2050 (O’Neill, 2016). Consequently, the demand to identify antimicrobial molecules that can overcome the resistance responses of bacteria is constant and challenging (Magana, 2020). Even when such molecules are identified, they must be formulated into medicines with adequate bioavailability to ensure efficacy upon administration to a patient. Antimicrobial peptides (AMPs) are a diverse group of small bioactive proteins found naturally in many living organisms as part of their immune system (Annunziato and Costantino, 2020; Guaní-Guerra et al., 2010). AMPs have been widely investigated for clinical applications, including bacterial infectious diseases (Guaní-Guerra et al., 2010). Bacteriocins are small, often cationic AMPs synthesized by ribosomes in bacteria (Balandin et al., 2019; Baindara et al., 2018; Meade et al., 2020). The difference in their amphiphilic helices gives bacteriocins distinct structures, spectra of activity, and biochemical properties (Meade et al., 2020).

Thuricin CD is a two-component bacteriocin produced by *Bacillus thuringiensis* DPC 6431, consisting of two post-translationally modified peptide chains, Trn α and Trn β, which have been shown to act synergistically against spore-forming gram-positive bacteria, including the most virulent strain of *Clostridioides difficile*, PCR ribotype 027/NAP-1 (Mathur, et al., 2017). *C. difficile*-associated disease (CDAD) is a common nosocomial infectious disease characterized by diarrhoea, abdominal cramps, and, in severe cases, pseudomembranous colitis, which can be life-threatening if left untreated or if the treatment fails (Rea, 2010). Unlike other bacteriocins and antibiotics previously used to treat this disease, thuricin CD exhibits narrow-spectrum activity that could help to prevent a detrimental impact on the commensal microbiota (Rea, 2010). Additionally, thuricin CD has shown activity against some *Listeria monocytogenes* strains (Rea, 2010). *L. monocytogenes* is a foodborne pathogen that especially affects immunocompromised people, the elderly, and pregnant women, causing meningoencephalitis and foetal-placental infection, respectively (Lecuit, 2020). Thus, thuricin CD shows potential to be used as a new treatment for *C. difficile* and *L. monocytogenes* infections.

However, because of their peptide nature, bacteriocins are susceptible to degradation, aggregation, and precipitation in the body, and can show low solubility and poor permeability (Flynn et al., 2021). Previous reports have evaluated the heat and pH stability of thuricin CD cell-free supernatants. Thuricin CD was stable at a temperature as high as 85 °C, reducing its activity when reaching 90 °C and exhibiting complete activity loss at 100 °C. Activity was also maintained in a pH range of 2 to 9 (Rea, 2010). Neither quantitative nor qualitative data on the solubility of thuricin in biorelevant media is available. Sit et al. have reported the hydrophobicity of Trn α and Trn β, whose hydrophobic residues pointing outwards explain the necessity of at least 50 % organic solvent with water to dissolve the peptides (Sit et al., 2011). Enzymatic degradation of thuricin CD is reported in one study (Rea, 2014). It is stated that Trn β was degraded by pepsin and α chymotrypsin *in vitro* as well as *in vivo*, while Trn α was resistant to both enzymes (Rea, 2014). Durack et al. suggest that to improve the stability and *in vivo* application of a particular bacteriocin, it is important to identify a vehicle that can protect and deliver the peptide as and where planned (Durack, 2019). Encapsulation technologies have been suggested as a viable alternative, particularly to prevent degradation (Naskar and Kim, 2021).

Lipid delivery systems have been widely used for many years to deliver drugs that treat severe infections, cancer, and recently, COVID-19 (Alghurabi et al., Jun. 2022; Gregoriadis, 2021). There is also research on the use of lipid systems for the delivery of antimicrobial drugs. Some of the advantages that these systems offer are biocompatibility, biodegradability, and the ability to incorporate hydrophobic and hydrophilic drugs (Ferreira, 2021; Lee et al., 2005). Moreover, liposomes loaded with antibiotics have shown increased antimicrobial activity compared to the free antibiotic (Ferreira, 2021). It is hypothesised that this could be due to the similarity of the liposomes with the bacterial membrane allowing them to interact extensively, fuse, and deliver higher concentrations of antibiotic (Ferreira, 2021). However, conventional liposomes have shown susceptibility to bile salts and enzymes present in the gastrointestinal tract (Lee et al., 2005).

Antimicrobial peptides have been previously formulated into liposomes for oral delivery. Vancomycin, for example, was encapsulated in distearoylphosphatidylcholine (DSPC)- cholesterol (CH)- diacetyl phosphate liposomes coated with folic acid, and lecithin-CH, glycer-ylcaldityltetraether liposomes (Anderson et al., 2001; Uhl, 2017). Bioavailability was increased by 12.5 and 3-fold respectively, compared with a vancomycin solution. Colistin was also loaded into lecithin-CH surfactant S60 liposomes coated with chitosan, and CH-dipalmitoyl-phosphatidylcholine/DSPC-1,2-dipalmitoyl-*sn*-glycero-3-phospho-ethanolamine-N-(Glutaryl) liposomes coated with an extracellular adherence protein (Aboumanei et al., 2021; Menina, 2019). Improvements in bioavailability was reported in the former, and a better anti-microbial effect in the latter liposome design. Several of these formulations involve the coating of liposome surfaces with polymers to improve the stability of the liposomes.

Another approach to produce more stable liposomes has been to modify the lipid composition by including saturated lipids with high transition temperatures (Kokkona et al., 2000). These lipid properties contribute to form more rigid liposomal bilayers that are less likely to be deformed (Lee et al., 2005). This last strategy has been applied in the work reported here.

The focus of this study was to, first, evaluate the bioactivity, susceptibility to proteases, solution behaviour in physiological media, and cytotoxicity of thuricin CD. Based on these results, an anionic lipid-based delivery system for thuricin CD was developed and the activity, release, enzyme stability in GI fluids, activity after exposure to GI fluids, and storage stability of the encapsulated peptides compared to the free and dissolved peptides were evaluated.

## 2 Materials and methods

### 2.1 Materials

*B. thuringiensis* DPC 6431 was provided by our collaborators at Teagasc Food Research Centre, Moorepark, Co. Cork, Ireland. *L. monocytogenes* culture (ATCC 1916) and CaCo2 cells were purchased from ATCC. LB broth, brain heart infusion (BHI) broth, XAD16N beads, ethanol, acetonitrile (ACN, ≥99.9 %), isopropanol (IPA, ≥99.9 %), phosphate-buffered saline (PBS), trifluoroacetic acid (TFA, ≥99.9 %), sodium hydroxide (NaOH), trypsin (1000–2000 BAEE units/mg), α-chymotrypsin (≥40 units/mg), pepsin (no specified activity units), hydrochloric acid (HCl, 36.4–38 %), L-α-phosphatidylcholine hydrogenated (soy, HSPC), 1,2-Dipalmitoyl-*sn*-glycero-3-phosphoglycerol, sodium salt (DPPG Na), sodium acetate, electron microscopy grade glutaraldehyde G5882, and acetic acid were all purchased from Sigma Aldrich Ireland Ltd. Chloroform, methanol (HPLC grade), sodium chloride (NaCl), Dulbecco’s Modified Eagle’s medium high glucose, fetal bovine serum, L-glutamine, non-essential amino acids, penicillin–streptomycin and Presto blue were purchased from Fisher Scientific Ireland. Sinapinic acid and peptide calibrant II were purchased from Bruker, Mannheim, Germany. Biorelevant FaSSIF/FaSSGF powder was purchased from Biorelevant.com Ltd. Deionised water was obtained from the Elga PURELAB System with a purity of 18.2 mΩ.cm.

### 2.2 Production and purification of thuricin CD

Thuricin CD was produced and purified according to the protocol developed by Rea et al. with minor modifications (Rea, 2010). Briefly, *B. thuringiensis* DPC6431 was cultured aerobically in BHI broth. This was partially purified by hydrophobic interaction chromatography by passing it through a column with XAD 16 N resin beads. Further purification of thuricin CD was carried out by solid phase extraction (SPE) with a Strata C18-E column. Thuricin CD peptides were separated by reverse-phase high-performance liquid chromatography (RP-HPLC), lyophilised in a Genevac HT4 lyophiliser, and stored at −20 °C. This lyophi-lized peptide was used for all further experiments. MALDI TOF mass spectrometry was performed on the purified thuricin CD peptides to confirm their purity (supplementary information).

### 2.3 RP-HPLC analysis of thuricin CD

Stock solutions of 1 mg/mL of freeze-dried Trn α and Trn β were prepared in 70 % IPA 0.1 % TFA in water. Further dilutions of each peptide to concentrations of 2.5–250 μg/mL were made up in 50:50 (v/v) 70 % IPA 0.1 % TFA in water: 0.1 % TFA in water (v/v). All samples were filtered through a 0.2-μm PES filter before being analysed by RP-HPLC on an Agilent 1260 Infinity system with ultraviolet (UV)-vis detection at 214 nm. The column used was an Aeris 3.6 μm peptide XB-C18 100 250 x 46 mm (Phenomenex, Cheshire, UK) with a flow rate of 0.8 mL/min. The mobile phase used was 0.1 % TFA in water as aqueous phase A and 0.1 % TFA in acetonitrile as organic phase B with a gradient of 35 to 85 % 0.1 % TFA in acetonitrile over 65 min. The peak area (mAU) was plotted against the concentration (μg/mL) to obtain a calibration curve. R^2^ values were 0.99999 for both Trn α and Trn β. This data was employed afterwards to quantitatively determine Trn α and Trn β concentrations.

### 2.4 Bioactivity of dissolved thuricin CD against L. monocytogenes ATCC 1916

Thuricin CD has previously shown activity against bacterial strains such as *C. difficile* (Rea, 2010). For this study, *L. monocytogenes* was used as an indicator strain. The minimum inhibitory concentration MIC_50_ was determined based on the protocol previously described by Mathur et al. (Mathur, et al., 2017). MIC_50_ was established as the concentration of peptides that inhibited 50 % of growth of *L. monocytogenes* ATCC 1916 (Rea, 2010).

Briefly, 1 mg/mL stock solutions of freeze-dried Trnα and Trnβ resuspended in 70 % IPA 0.1 % TFA in water were diluted in sterile PBS to a final concentration of 100 μg/mL (dissolved thuricin). For all the experiments in this, and the following sections, a 1:1 M ratio of Trn α: Trn β was used. The corresponding volumes of each peptide to reach 250 nM of each peptide (0.69 μg/mL Trn α + 0.72 μg/mL Trn β), 500 nM of each peptide (1.38 μg/mL Trn α + 1.43 μg/mL Trn β), 1 μM of each peptide (2.76 μg/mL Trn α + 2.86 μg/mL Trn β), and 2 μM of each peptide (5.53 μg/mL Trn α + 5.72 μg/mL Trn β) in the final well volume were added in triplicate to a 96 well plate. A volume of 50 μL was reached in each well by adding PBS before addition of the bacterial culture. *L. monocytogenes* ATCC 1916 was grown in BHI broth shaking overnight at 37 °C. 150 μL of 10^5^ CFU/mL bacterial culture was added to the wells with sample. Blanks were set up in triplicate with only PBS and media as well as a control containing buffer and bacterial culture. The plate was incubated in a Biotek ELx808 Ultra microplate reader at 37 °C for 24 h. Readings were taken every 30 min at 590 nm. The same procedure was carried out for testing Trn α or Trn β alone at concentrations ranging from 2 to 5 μM against *L. monocytogenes*.

### 2.5 Enzymatic degradation of dissolved thuricin CD

Trn α and Trn β stock solutions (1 mg/mL in 70 % IPA 0.1 % TFA in water) were filter sterilized and diluted to a final concentration of 100 μg/mL in sterile PBS. Pepsin activity was evaluated beforehand according to the haemoglobin assay, based on the principle that haemoglobin in water produces tyrosine peptides soluble in trichloroacetic acid (TCA) when pepsin is applied (Brodkorb, 2019). The activity units (AU or μmol/min), defined as the quantity of the enzyme that converts one micromole of substrate per minute, were determined before this test. Pepsin was suspended in cold 0.01 M HCl pH 2 reaching 824 activity units per mL. Trn α and Trn β were added to the same well of a 96-well plate in triplicate to give a final concentration of 5.6 μg/mL and 5.8 μg/mL respectively. This concentration had been shown to be sufficient to show activity against a *L. monocytogenes* culture. 25 μL of pepsin solution was also added to each of the wells. This gave a final pepsin concentration/activity of 1.2 mg/mL or 98.8 AU/mL, which is the approximate concentration of enzyme present in the human stomach (Liu, 2015). Thuricin alone and pepsin alone were included as control samples with the bacterial culture. Blanks were set up in triplicate with PBS, 0.01 M HCl, and media only (BHI). The plate was incubated for 1.5 h at 37 °C, 70 rpm to allow the enzyme to act. After incubation, each well was filled with 150 μL of diluted bacterial cell culture (10^5^ CFU/mL *L. monocytogenes*). The plate was incubated in a BioTek ELx808 Ultra microplate reader at 37 °C for 24 h at a wavelength of 590 nm. Readings were taken every 30 min for 24 h with the plate shaken mildly before each reading.

A similar procedure was followed for trypsin and chymotrypsin, but different concentrations were used, and incubation time was 3 h. Trn α and Trn β were added to a final concentration of 4.4 μg/mL each. Trypsin solution of 131.1 BAEE units /mL in water was used as well as chymotrypsin solution of 6.2 AU/mL in water. Trypsin activity units were determined by a previously established assay based on the principle that p-toluene-sulfonyl-l-arginine methyl ester (TAME) in water produces p-toluene-sulfonyl-L-arginine plus methanol when trypsin is applied (Brodkorb, 2019). Chymotrypsin activity was evaluated with a test based on the principle that N-Benzoyl-L-tyrosine ethyl ester (BTEE) in water produces N-Benzoyl-L-tyrosine plus ethanol when chymotrypsin is applied (Brodkorb, 2019).

### 2.6 Solution behaviour of free thuricin CD in fasted state simulated gastric and intestinal fluid without enzymes

Fasted state simulated intestinal fluid (FaSSIF) and fasted state simulated gastric fluid (FaSSGF) were prepared using Biorelevant FaS-SIF/FaSSGF powder following the manufacturer’s instructions. Briefly, for FaSSIF, 0.21 g NaOH, 1.72 g sodium phosphate monobasic and 3.10 g NaCl were diluted in 400 mL distilled water. The solution was stirred until dissolved. The pH was then adjusted to 6.5 with 0.1 M HCl. 1.12 g FaSSIF powder was added and finally, the volume was topped up to 500 mL. For FaSSGF, 2 g NaCl was dissolved in 900 mL distilled water. The pH was adjusted to 1.6 with 1 M HCl. 0.06 g of FaSSGF powder was added and the volume was completed to 1 L. The final compositions are shown in Table 1.

Trn α and Trn β solutions at concentrations of 0.5 mg/mL in FaSSGF/FaSSIF were suspended directly from lyophilised powder (free thuricin). Further dilutions were carried out to reach concentrations of 2 times, 5 times and 10 times the MIC_50_ in the final volume to be used for the experiment. The suspensions were distributed in different microtubes and incubated at 37 °C, 300 rpm in an incubator for 0.5 h. An aliquot of each concentration was taken, and filter sterilized with a 0.2 μm PES filter. 50 μL of each sterile solution was transferred to a 96-well microtiter plate in triplicate. Each well was filled with 150 μL of a *L. monocytogenes* culture at an OD of 0.1. A control was set with 50 μL of FaSSGF/FaSSIF and 150 μL culture. The plate was incubated in a Biotek ELx808 Ultra microplate reader at 37 °C for 24 h at a wavelength of 590 nm. Readings were taken every 30 min for 24 h with the plate shaken mildly before each one. The readings collected from those samples were considered as time 0. In the meantime, the microtubes were put back in the incubator with reduced shaking. After 1, 4 and 24 h a new aliquot was taken, and the same protocol was followed for activity testing.

### 2.7 Trn αβ liposomes

#### 2.7.1 Preparation of Trn αβ liposomes

L-α-phosphatidylcholine hydrogenated (soy, HSPC) and 1,2-Dipal-mitoyl-*sn*-glycero-3-phosphoglycerol, sodium salt (DPPG) liposomes were prepared by thin film rehydration. 25 mg of each lipid, HSPC and DPPG, were dissolved in 5 mL chloroform: methanol 2:1 (v/v) in a round-bottom flask. Trn α and Trn β were also added in this step directly from freeze-dried powders at a concentration of 0.1 mg/mL in the final suspension. The organic solvents were removed using a rotavapor for one hour at 60 °C under vacuum to obtain a thin lipid film. The film was left to dry under N_2_ for 30 min to eliminate any remaining organic solvent. Subsequently, 5 mL acetate buffer (pH 4, 0.1 M) was added to rehydrate the thin film at 60 °C with stirring. Tip-sonication at an amplitude of 40 %, pulse on 15 s and pulse off 15 s, for 10 min was used to reduce the size of multilamellar vesicles with a Qsonica CL-18 4422 sonicator probe.

#### 2.7.2 Storage stability of Trn αβ liposomes

The storage stability of the Trn αβ liposome suspension in acetate buffer was measured over 38 days at room temperature and 4 °C by monitoring size, polydispersity index (PDI) and ζ-potential through dynamic light scattering (DLS). For size measurement, the sample was diluted 1:100 (v/v) in DI water and placed in a Malvern Nano ZetaSizer in a disposable plastic cuvette. The material selected was lipid, with a refractive index of 1.5, and the dispersant was water, with a refractive index of 1.33. For zeta potential, the same instrument and parameters were used but in a disposable folded capillary cell.

#### 2.7.3 Encapsulation efficiency of Trn αβ liposomes

The encapsulation efficiency of thuricin CD into the liposomes was investigated by determining the total peptide (TP) content, encapsulated peptide (EP) content, and free peptide (FP) content. The TP content was analysed by disrupting the liposomes in the thuricin liposome suspension with 1:1 (v/v) chloroform: methanol at a ratio of 1:10 (v/v). The solution was vortexed and then sonicated in a water bath for 10 min. The solution was filtered with a 0.2 μm PES filter and analysed by our previously developed reverse phase high-pressure liquid chromatography (RP-HPLC) method. For the EP and FP content, thuricin liposomes were first separated from free peptide by ultrafiltration. The liposome dispersion was loaded to an Amicon Ultra-0.5 MWCO 30 K centrifugal filter unit and centrifuged at 6700 rcf for 15 min. For EP, the separated liposomes were resuspended in acetate buffer. Then, this liposome suspension was diluted in 1:1 (v/v) chloroform: methanol at a ratio of 1:10 (v/v). The solution was vortexed and filtered with a 0.2 μm PES filter. EP and FP fractions were analysed by RP-HPLC. A control with blank liposomes (no encapsulated thuricin) was prepared following the same method as for TP analysis. As a control experiment, a sample of blank liposomes were disrupted the same way as for TP analysis and spiked with 10 μg/mL Trn α + 10 μg/mL Trn β.

#### 2.7.4 Release of thuricin CD from Trn αβ liposomes

A 6,000 Da cut off mini dialysis tube was filled with 765 μL of Trnαβ liposome suspension (100 μg/mL Trnα and 100 ug/mL Trnβ) and added to a glass vial with 4.5 mL of FaSSIF/ FaSSGF. Samples were stirred at 100 rpm in a plate at 37 °C. At 1 and 3 h, 0.5 mL of FaSSGF release media were removed, filtered with a 0.2 μm PES filter, and analysed by our previously described RP-HPLC method. FaSSIF release media samples were taken at 1, 3, 6, 24, and 72 h.

#### 2.7.5 Biological activity of Trn αβ loaded liposomes vs free thuricin CD in acetate buffer

The activity of Trn αβ liposomes was evaluated following the methodology previously described for thuricin CD. Briefly, the liposome suspension was filtered with a 0.45 μm PES sterile filter. Different volumes of the sterile liposome suspension were added to reach final concentrations of Trn α and Trn β of 1.25 and 2.50 μg/mL in each well. Samples were tested in triplicate. A volume of 50 μL was reached in each well by adding PBS. 150 μL of 10^5^ CFU/mL *L. monocytogenes* ATCC 1916 culture was added to the wells with sample. Blanks were set up in triplicate with only media, the corresponding blank liposome suspension volume, and PBS. Controls contained inoculated media and PBS. The plate was incubated in a BioTek ELx808 Ultra microplate reader at 37 °C for 24 h. Readings were taken every 30 min at 590 nm. The same procedure was followed for free thuricin (freeze dried thuricin powder directly suspended in acetate buffer).

#### 2.7.6 Study of the effect of Trn αβ loaded liposomes vs dissolved thuricin CD on bacterial cell morphology by SEM imaging

SEM images of bacteria after treatment with Trn αβ loaded liposomes and dissolved thuricin CD peptides were obtained according to a previously developed method (Ratrey et al., 2020). Briefly, an overnight *L. monocytogenes* culture was diluted to an OD_595_ reading of 0.2 and incubated for 24 h at 37 °C with Trn αβ loaded liposome suspension or dissolved thuricin (thuricin powder in 70 % IPA 0.1 % TFA in water and further diluted in PBS) at a concentration of double the observed MIC_50_. Controls were set with blank liposome suspension and PBS. After incubation, the samples were centrifuged at 300 rcf for 7 min. The supernatant was removed, and the pellet was fixed with 2.5 % glutaraldehyde in PBS overnight at 37 °C. Glutaraldehyde was removed, and the pellet was washed once with PBS and three times with sterile water. This was then freeze-dried in a Telstar Lyoquest freeze dryer at – 80 °C and 0 mbar. The dried cells were placed on a sticky carbon disc onto a metal stub and coated with gold for 120 s at 20 mA in an Emitech K55 system. Images of these *L. monocytogenes* cells treated with Trn αβ liposomes and dissolved thuricin CD were obtained by SEM on a Hitachi SU-70.

#### 2.7.7 Size stability of Trn αβ liposomes in FaSSGF and FaSSIF with enzymes

The stability of Trn αβ liposome suspension after being exposed to gastrointestinal fluids with enzymes was evaluated based on a previously developed method with minor alterations (Ryan, Jul. 2022). Fasted state simulated intestinal fluid (FaSSIF) and fasted state simulated gastric fluid (FaSSGF) were prepared as described in a previous section, following the manufacturer’s instructions. Pepsin was added at a concentration of 10 mg/mL to FaSSGF and pancreatin at 2.1 mg/mL to FaSSIF just before the experiment. The volumes necessary to reach concentrations of 25 μg/mL Trn αβ liposomes + 1.2 mg/mL (98.8 AU/mL) pepsin in FaSSGF were added to a microtube and filled to 500 μL with FaSSGF. A control with the same proportion of αβ liposomes was prepared in water. Samples were incubated at 37 °C with 70 rpm stirring. At 15 and 30 min a sample was removed from FaSSGF and water before undergoing analysis by DLS.

The same procedure was followed for αβ liposomes in FaSSIF with pancreatin but with a final concentration of the enzyme of 1.6 mg/mL, and with an incubation time of 3 h.

#### 2.7.8 Biological activity of Trn αβ liposomes vs dissolved peptide after incubation in FaSSGF with pepsin and FaSSIF

The activity of αβ liposome suspension and dissolved αβ peptides after exposing both to FaSSGF with pepsin and FaSSIF without pancreatin was analysed. No pancreatin was added to FaSSIF because it inhibitied *L. monocytogenes* growth itself. Trn αβ loaded liposome suspension or dissolved thuricin (thuricin powder in 70 % IPA 0.1 % TFA in water and further diluted in acetate buffer) + pepsin in FaSSGF were prepared as described in the previous section. Control samples with acetate buffer + pepsin in FaSSGF were also prepared.

After 30 min incubation, pepsin was deactivated by heating the samples at 70 °C for 10 min. The volume necessary to reach a concentration of double the MIC_50_ of thuricin peptides was added to a 96-well plate. The wells were topped to 50 μL with PBS. 150 μL of 10^5^ CFU/mL bacterial cell culture (*L. monocytogenes*) was added to each well. Blanks were set with media and control samples. The plate was incubated in a BioTek ELx808 Ultra microplate reader at 37 °C for 24 h at a wavelength of 590 nm. Readings were taken every 30 min for 24 h with the plate shaken mildly before each reading.

The biological activity of Trn αβ liposome suspension and dissolved peptide after exposure to FaSSIF was also evaluated by total plate counts. A concentration of 11.2 μg/mL Trn α + 11.2 μg/mL Trn β in Trn αβ liposome suspension and dissolved thuricin CD (thuricin powder in 70 % IPA 0.1 % TFA in water and further diluted in acetate buffer) was added to a microtube with FaSSIF in a total volume of 300 μL and incubated shaking at 70 rpm, 37 °C for 3 h. A control with acetate buffer and FaSSIF was incubated at the same conditions. 50 μL of sample were added to a 96 well plate with 150 μL of 10^5^ CFU/mL *L. monocytogenes* ATCC 1916 culture to reach a concentration of double MIC_50_ of thuricin. The plate was incubated at 37 °C for 24 h with mild shaking every 30 min. All samples were added in triplicate with the corresponding blanks. Samples were diluted in PBS by decimal dilution factors as needed, and 10 μL were added by drops in a BHI agar plate. All plates were incubated inverted overnight at 37 °C. The colony forming units (CFU) per mL were counted and compared to the control samples.

#### 2.7.9 Cytotoxicity of Trn αβ liposomes vs dissolved thuricin CD

The biocompatibility of Trn αβ liposome suspension and dissolved thuricin CD with intestinal epithelial cells were tested. Caco-2 cells were grown in high glucose Dulbecco’s modified Eagle’s medium supplemented with 10 % fetal bovine serum, 1 % L-Glutamine, 1 % pen-strep, and 1 % non-essential amino acids at 37 °C, with 5 % CO_2_ and 95 % relative humidity. Approximately 10,000 cells were seeded per well in a 96-well plate. The plate was incubated overnight to ensure the cells adhered to the wells. The media was removed from the wells and filter-sterilized dissolved thuricin CD and Trn αβ liposome suspension samples at a final concentration of 5 μg/mL of each peptide were added 1:1 (v/v) with fresh media. Control wells were included by adding blank liposomes and just media to the cells. The plate was incubated for 24 h at 37 °C, with 5 % CO_2_ and 95 % relative humidity. The media was removed from the wells, 10 μL Presto blue +90 μL fresh media was added to each well and left incubating for 60 min. Finally, the absorbances of the solutions in the plate were read at 570 nm and plotted for all samples.

##### 2.7.9.1 Statistical analysis

A one-way ANOVA test was used to determine significant differences in the biological activity of Trn αβ liposomes vs dissolved peptide after incubation with FaSSIF. Tukey’s and Levene’s tests were used to compare the mean values and variances, respectively, at a probability threshold of 0.05. A value of p ≤ 0.05 was considered significantly different for both tests.

## 3 Results and discussion

### 3.1 Purification, and characterisation of thuricin CD

Trn α and Trn β peptides were purified with a varying yield of 3 to 8 mg/L. Trn α and Trn β fractions were collected from the semi-preparative RP-HPLC stage and freeze-dried before resuspension or encapsulation into liposomes as described in the methods section. The molecular mass of the individual peptides was confirmed by MALDI mass spectroscopy (supplementary information).

### 3.2 The bioactivity of dissolved thuricin CD against L. Monocytogenes ATCC 1916

**Fig. 1:**
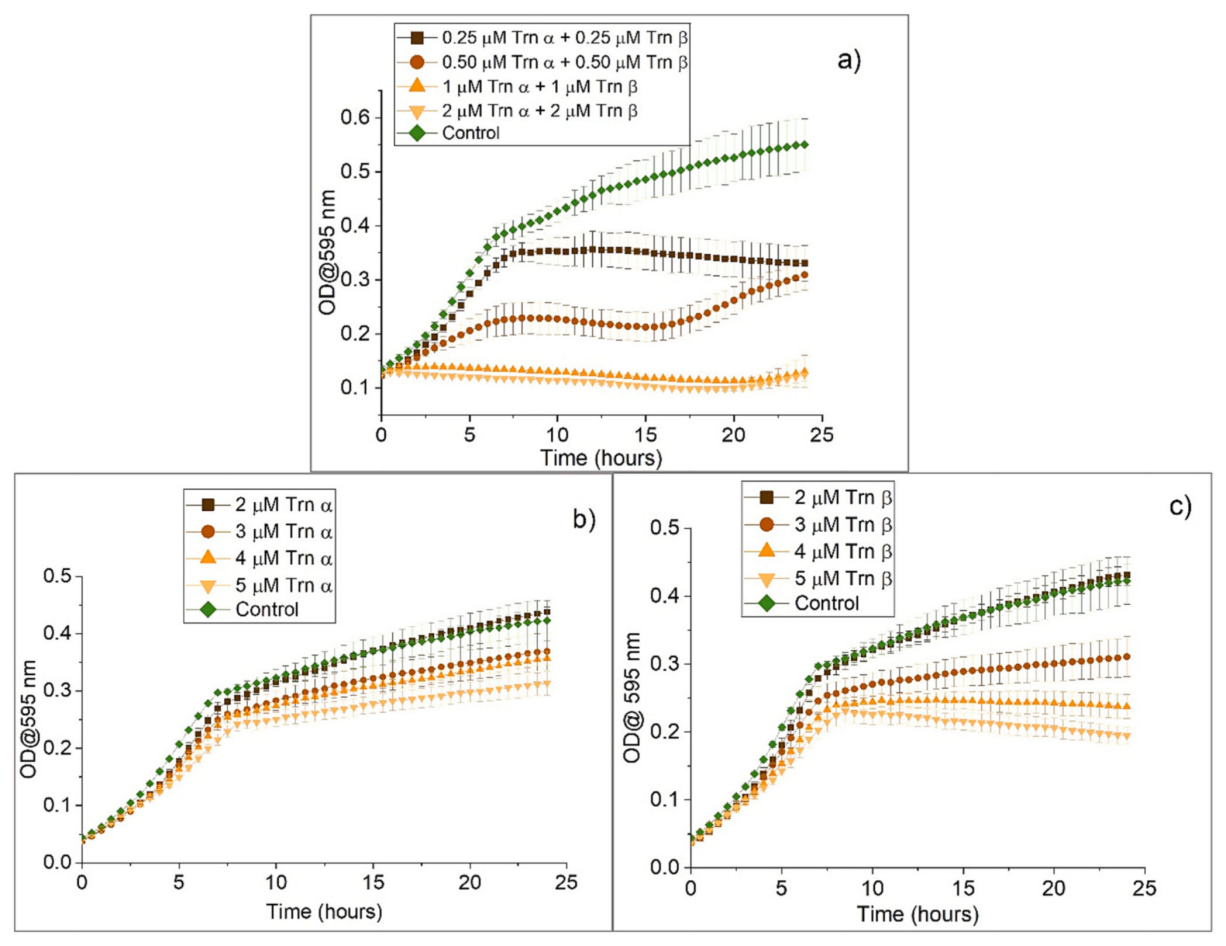
Activity of a) both thuricin CD peptides (at a 1:1 M ratio of Trn α: Trn β), and the individual peptides b) Trn α and c) Trn β against L. monocytogenes ATCC 1916 over 24 h at 37 ° C at a range of peptide concentrations. The control refers to the samples containing only PBS and bacteria.”.

Previous work has reported the sensitivity of some *L. monocytogenes* strains (DPC 1786, DPC 3437, Scott A) to thuricin CD (Rea, 2010). Thus, the antibacterial effect of the peptides produced in-house against the bacterial strain *L. monocytogenes* ATCC 1916 was determined at specific concentrations. This set the basis of the concentrations to be used for subsequent characterisation and formulation evaluation. From Fig. 1a, a concentration of 1 μM of each peptide (2.76 μg/mL Trn α + 2.86 μg/mL Trn β) maintained the same OD (0.1) for 24 h, meaning growth of *L. monocytogenes* was inhibited at this concentration. A concentration of 0.5 μM of each peptide shows inhibition of bacterial growth by approximately 50 %. Thus, concentrations of 1.38 μg/mL Trn α + 1.43 μg/mL Trn β were set as the MIC_50_. A concentration of 0.25 μM of each peptide of thuricin CD could not significantly inhibit bacterial growth, however, it does show a slight reduction. None of the individual peptides alone, Trn α nor Trn β, could completely inhibit growth up to concentrations as high as 5 μM (Fig. 1b, c respectively). These results confirm that the amount of Trn α and Trn β needed to inhibit *L. monocytogenes* is very low when used together and that they act synergistically. Previous studies have shown that the concentrations of thuricin CD needed for inhibiting bacterial growth are much lower than those of each individual peptide (Mathur, et al., 2017). It is important to note that the results obtained in this section correspond to the activity of thuricin CD which was dissolved in 70 % IPA 0.1 % TFA in water before dilution with PBS. IPA and TFA were required to ensure dissolution of the peptides.

### 3.3 Enzymatic degradation of dissolved thuricin CD

The susceptibility of peptide drugs to proteolytic enzymes is one of their main disadvantages (Radaic et al., 2020). Previous studies have reported the susceptibility of Trn β to degradation when exposed to pepsin, while Trn α was resistant to digestion by the same enzyme (Rea, 2014). In that work, both peptides were treated with pepsin and left to act for 2 h. After that time, MALDI TOF mass spectrometry was used to detect the presence or absence of each peptide (Rea, 2014). The same work describing susceptibility of thuricin CD to pepsin, reported the degradation of Trn β when exposed to chymotrypsin, while Trn α was resistant to the same enzyme (Rea, 2014). Oral delivery is the current aim of this study, due to its convenience and due to the disease the thuricin peptides are intended to treat, CDAD. Thus, the degradation of thuricin CD by enzymes present in the gastrointestinal tract (GIT) must be confirmed. Three proteases, pepsin, trypsin, and chymotrypsin were incubated with thuricin CD to confirm its susceptibility.

The enzymatic activity of the pepsin that was used for this assessment was 82.4 ± 5.4 U/mg using the haemoglobin assay (Brodkorb, 2019). Unfortunately, this value could not be compared to a theoretical one as the supplier did not provide any reference value. The enzymatic activity of the trypsin and chymotrypsin that was used for this section was 851.78 ± 29.58 BAEE units/mg and 40.9 ± 0.5 units/mg, respectively, compared to 1000–2000 BAEE units/mg and ≥ 40 units/mg which were the values provided by the suppliers respectively.

**Fig. 2:**
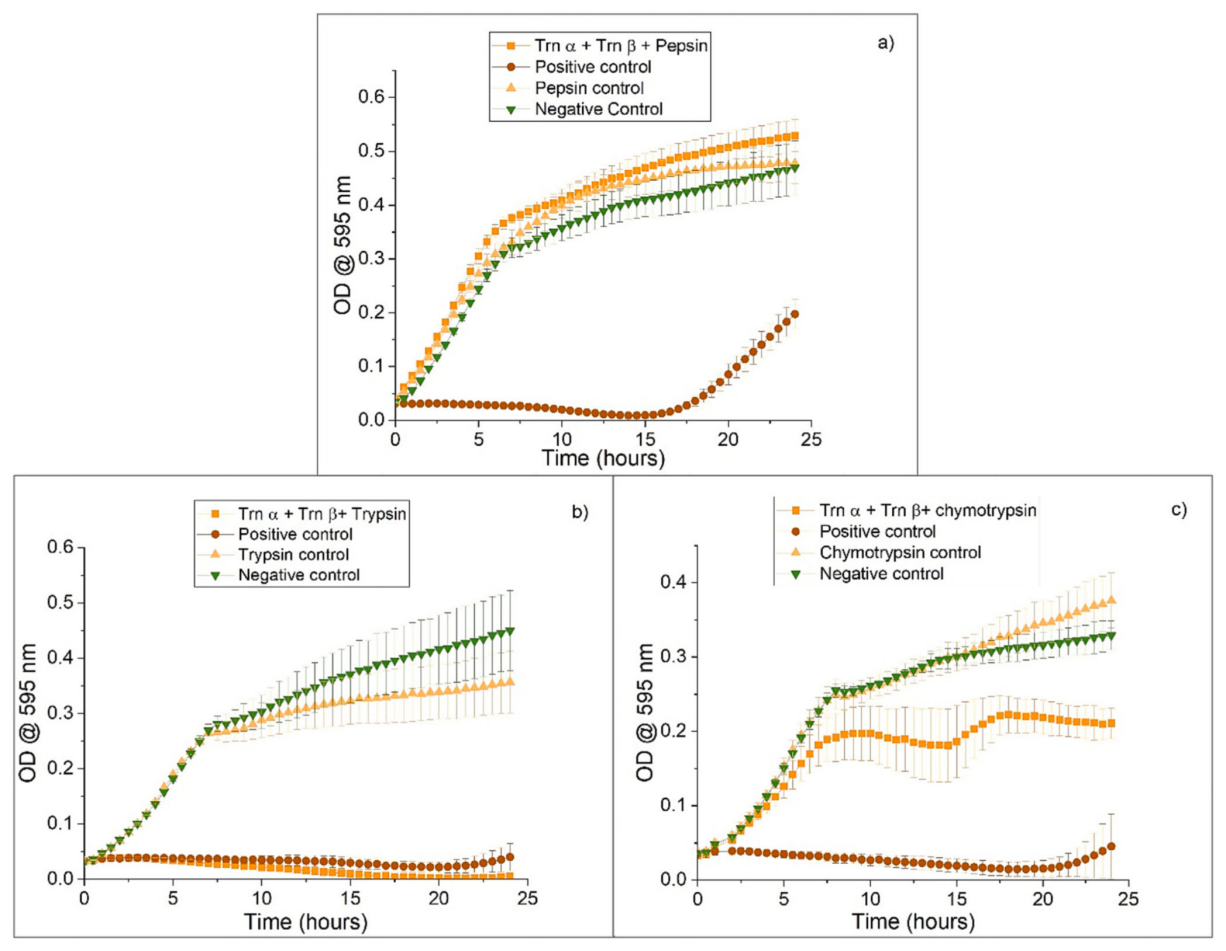
Activity of a) 5.5 μg/ml Trn α + 5.7 μg/ml Trn β b, c) 4.4 μg/ml Trn α + 4.4 μg/ml Trn β against L. monocytogenes ATCC 1916 after exposure to a) 1.2 mg/ml pepsin, b) 19.3 μg/ml trypsin, and c) 19.3 μg/ml α-chymotrypsin as an evaluation of degradation of the peptide with each enzyme. Positive control refers to the same concentration of peptides without enzyme. Negative control contains only PBS and bacteria. A control with just enzyme is added to check that it will not affect normal bacterial growth. Experiment was performed over 24 h at 37 °C.

The OD measurements from the well corresponding to the control show normal bacterial growth. Trn α and Trn β exposed to pepsin is the same as the one for the control, indicating that pepsin had deactivated the thuricin CD (Fig. 2a). The enzyme alone did not affect bacterial growth. On the other hand, the sample with thuricin alone inhibited bacterial growth for almost 17 h. Thus, pepsin degrades thuricin, affecting its activity against *L. monocytogenes* ATCC 1916. It is known that pepsin cleaves peptide bonds located within adjacent aromatic residues (Flynn et al., 2021). Trn α does not contain adjacent aromatic residues, while Trn β contains adjacent tyrosine and phenylalanine at sites 28 and 29 at the C-terminus (Sit et al., 2011). Pepsin is most likely to be cleaving between those residues and degrading Trn β. As both peptides are needed for activity at these concentrations, the peptides show no inhibition of bacterial growth after incubation with pepsin.

Fig. 2b shows that there was no growth in the wells containing Trn α and Trn β exposed to trypsin, just as there was in the wells with the thuricin CD peptide and no trypsin. Conversely, from Fig. 2c, Trn α and Trn β exposed to chymotrypsin showed growth comparable to the control. The enzyme alone does not affect bacterial growth. The sample with thuricin alone showed no growth as expected. It is known that trypsin cleaves arginine and lysine amino acids at the C-terminus (Flynn et al., 2021). Neither Trn α nor Trn β contain arginine or lysine amino acids at the C-terminus, so trypsin cannot affect any of the peptides (Sit et al., 2011) and thus thuricin CD retained its activity in the presence of trypsin. A-chymotrypsin cleaves proteins at the C-terminus of large hydrophobic residues such as leucine, phenylalanine, tyrosine, and tryptophan (Flynn et al., 2021). Trn β has a tyrosine residue at the C-terminus, which might be allowing chymotrypsin to cleave and degrade the peptide, affecting the overall activity of thuricin CD against *L. monocytogenes* ATCC 1916. This information confirms the importance of the development of a delivery system that could protect the peptide chains from degradation.

### 3.4 Activity of free thuricin CD in gastrointestinal media

One of the conditions for therapeutic activity of a peptide is its solubility in physiological media. Depending on the properties of the bacteriocin such as isoelectric point, hydrophobicity/hydrophilicity, amino acid composition and its structure, the pH or composition of the physiological media could render the bacteriocin insoluble, and thus inactive (Flynn et al., 2021). Gastrointestinal fluids are an important physiological location to consider as the initial aim of this investigation is to deliver thuricin orally for local activity in the intestine. Intestinal fluid pH is around 6.5 and gastric fluid pH is around 1.6. Neither quantitative nor qualitative data on the solubility of thuricin in bio-relevant media has been reported previously. The activity of thuricin CD against *L. monocytogenes* was measured when added to a FaSSIF solution (pH 6.5) and FaSSGF (pH 1.6) at concentrations of double, five and ten times the MIC_50_ concentration. Unlike the MIC experiments, where thuricin CD peptides were initially dissolved in 70 % IPA 0.1 % TFA in water, filtered, and then diluted to the target concentration in PBS, for these experiments, lyophilised thuricin CD powders were directly added into the physiological fluids under evaluation. Suspensions were obtained at all concentrations in all fluids.

**Fig. 3:**
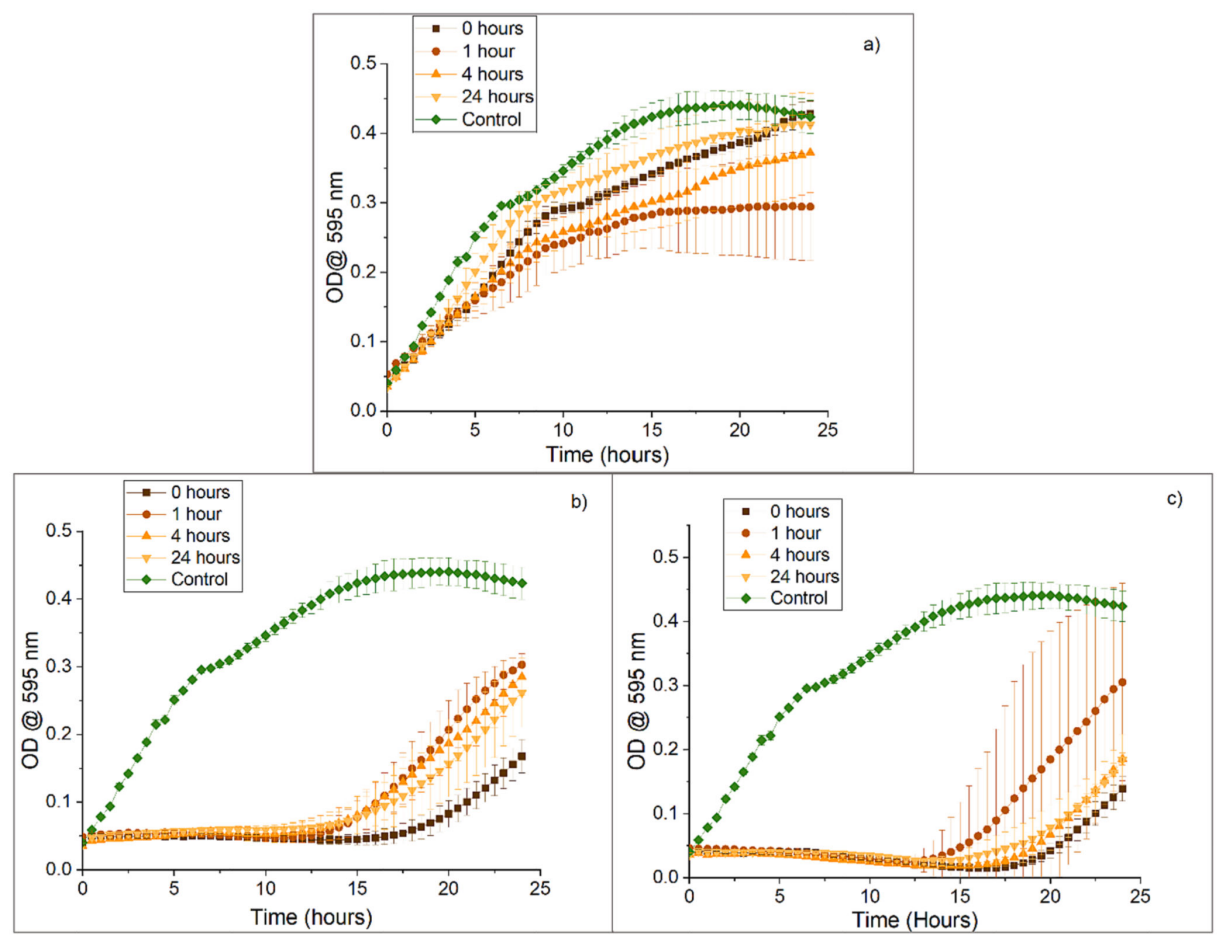
Activity of thuricin CD against *L. monocytogenes* ATCC 1916 after addition of lyophilised peptide powder to FaSSIF (pH 6.5) a) double the MIC50, b) five times the MIC50, and c) 10 times the MIC50.

When thuricin CD lyophilised powder is added to FaSSIF at concentrations of double the MIC_50_, no activity is observed (Fig. 3a). Nevertheless, activity is observed when the concentrations added were five and ten times the MIC_50_ (Fig. 3b and 3c respectively) for all time points (0–24 h). The pH in FaSSIF is 6.5, and the pI of Trn α and Trn β is close to 4, so the thuricin CD peptides should both be slightly negatively charged in this solution (ExPASy, 2022), helping their dissolution in this medium. Also, it has been previously reported that lacticin 3147, a two peptide bacteriocin with similar characteristics to thuricin CD, had improved solubility in FaSSIF compared to PBS (Ryan et al., 2021). It was hypothesised that this was due to micelles being formed as the concentration of sodium taurocholate (NaTC) in FaSSIF which was 3 mM, was above the critical micelle concentration (CMC) value for a 4:1 NaTc: phospholipid solution, 0.25 mM. The presence of micelles in FaSSIF alone was confirmed by DLS and it was also seen that, when each peptide was added to the solution, the size of micelles increased. This could mean that peptides were being trapped in the micelles because of hydrophobic interactions (Ryan et al., 2021). Based on this, it is hypothesised here that thuricin CD is entrapped in micelles that are being formed in the FaSSIF. Because of this, a stabilisation of activity can be seen above a concentration of five times the MIC_50_ in FaSSIF.

**Fig. 4:**
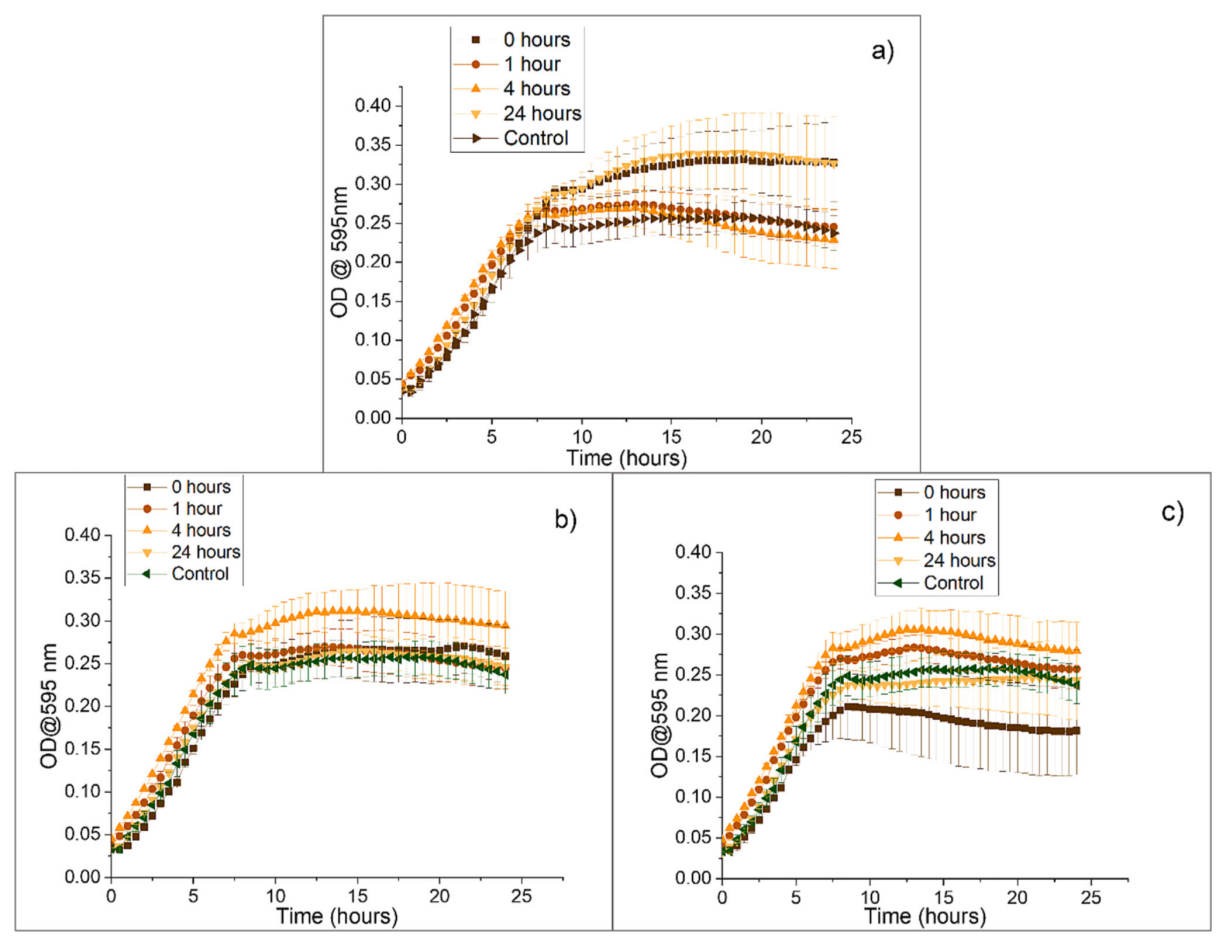
Activity of thuricin CD against *L. monocytogenes* ATCC 1916 after addition of lyophilised peptide powder to FaSSGF pH 1.6 for 0, 1, 4, and 24 h at concentrations a) x2 MIC50, b) x5 MIC50, and c) x10 MIC50.

In FaSSGF, no activity was detected for any peptide concentration (Fig. 4). This could be attributed to the hydrophobicity of thuricin CD, which is preventing it from dissolving. This was confirmed by the fact that, pre-dissolving the peptides in 70 % IPA 0.1 % TFA in water before diluting them in FaSSGF, retained thuricin CD activity (supplementary information). Glutamic acid residues govern the surface potential of Trn α and Trn β (Sit et al., 2011). The pH of FaSSGF is acidic (1.6), and at this pH, these residues would remain protonated, reducing its overall charge and polarity which would reduce the solubility in the gastric fluid.

### 3.5 Trn αβ loaded liposomes

The initial hydrodynamic size of the Trn αβ liposomes suspension in acetate buffer was 103.3 ± 0.7 nm, with a PDI of 0.21 and a zeta potential of −46.0 ± 3.8 mV. Previous studies have reported the size of HSPC-DPPG liposomes to be in the range of 100–130 nm, with a PDI of 0.2 and a zeta potential of −55.4 mV (Rinaldi, 2021);(Zhang et al., 2021). The negative charge of HSPC-DPPG liposomes is attributed to the presence of the anionic lipid DPPG Na.

**Fig. 5:**
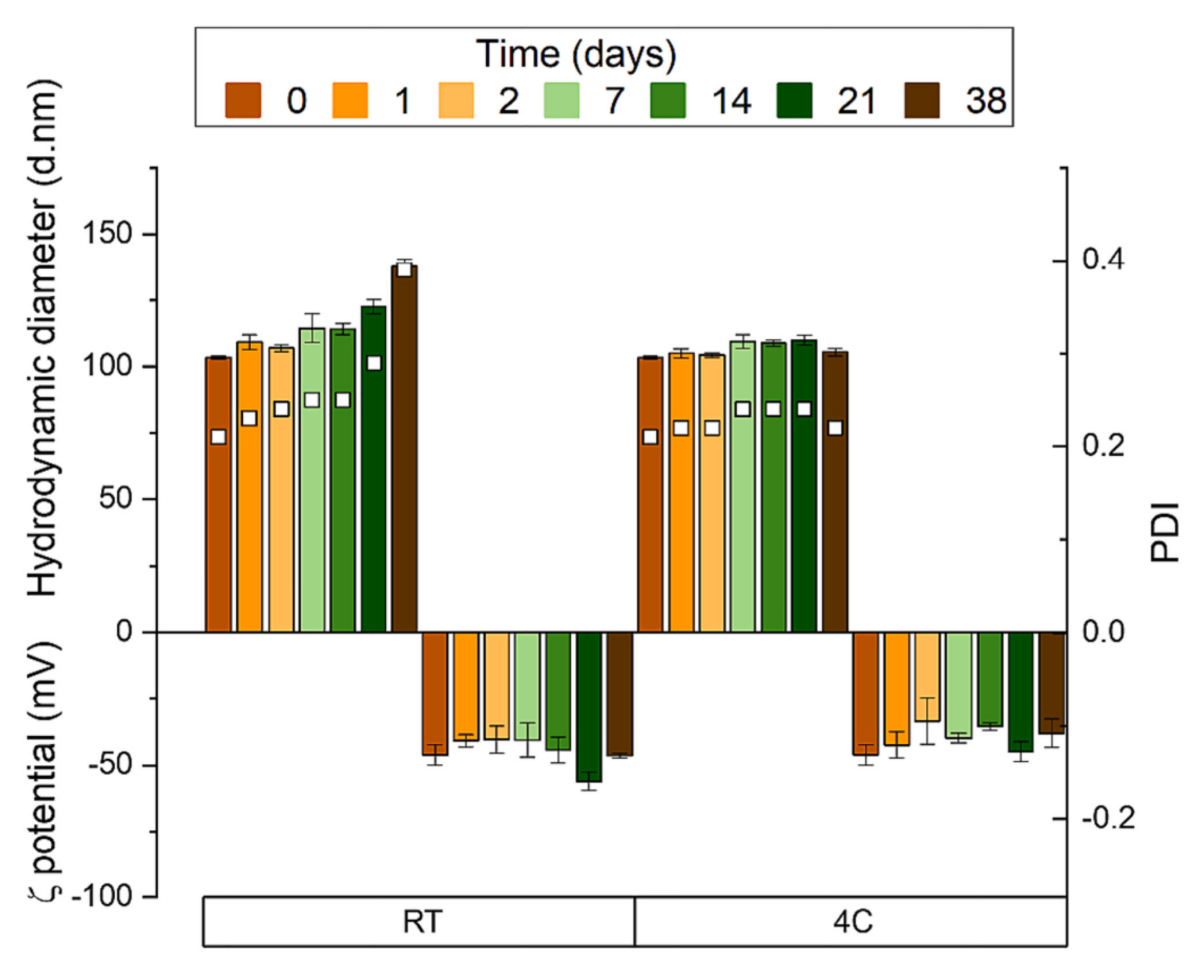
Stability of Trn αβ liposomes over 38 days at different storage temperatures (4 °C and room temperature), measured by size (top, coloured bars), PDI (top, white squares), and zeta potential (bottom, coloured bars).

Fig. 5 shows that the hydrodynamic diameter, PDI, and zeta potential of the thuricin-loaded liposomes stored at 4 °C did not significantly change over the course of 38 days, indicating their stability. This could be due to the negative zeta potential values, which suggest the presence of repulsive forces that prevent precipitation or aggregation. Earlier publications have described similar behaviour for anionic liposomes, which maintain stability for several days (Rinaldi, 2021). Liposomes stored at room temperature showed a stable hydrodynamic diameter, PDI, and zeta potential over 14 days, with slight increases in size and PDI on days 21 and 38, potentially indicating the start of aggregation. After 60 days stored at 4 °C, the liposome size was found to be 118.9±1.2 and the PDI was 0.3. Liposomes stored at room temperature were just inspected visually, where at longer time points a gel was formed, which confirmed aggregation and loss of stability.

**Fig. 6:**
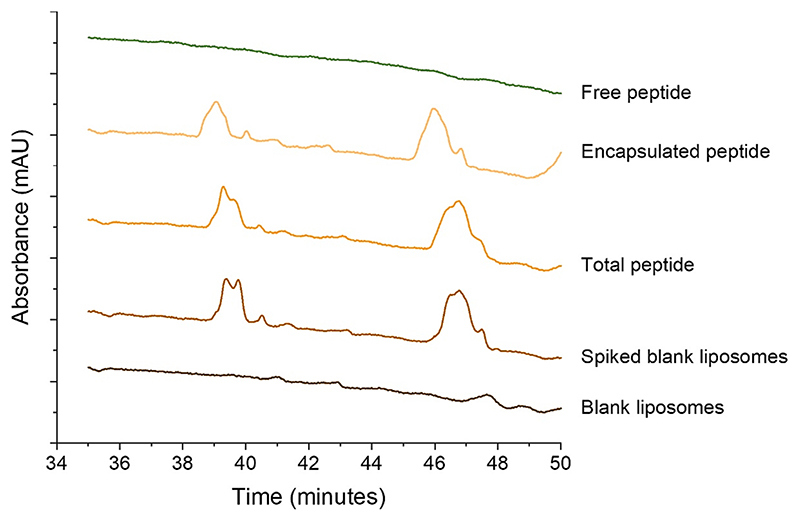
Chromatogram results for free, encapsulated, and total peptide from Trn αβ liposomes with spiked blank liposomes (disrupted blank liposomes with 10 μg/mL Trn α + 10 μg/mL Trn β added) and blank liposomes control.

We attempted to measure the encapsulation efficiency of thuricin CD in the anionic liposomes by analysing total, encapsulated, and free peptide content by RP-HPLC. Blank liposome samples were also incorporated as a control. It was seen in the chromatogram from the blank liposomes sample that no peaks are displayed at the elution times for Trn α and Trn β which are 40 and 47 min respectively. Similarly, the free peptide sample did not show peaks for either Trn α or β. The total peptide chromatogram showed broad peaks at the elution times corresponding to Trn α and β, Fig. 6. In the same way, broad peaks were obtained at Trn α and β elution times in the encapsulated peptide chromatogram. It was hypothesised that the lipids used for the liposomes are interacting with the peptide and interfering with the detection of the total peptide content by HPLC. To confirm this hypothesis, a suspension of blank liposomes was disrupted and spiked with Trn α and β, and then vortexed, sonicated, and filtered before analysis by HPLC. The chromatogram showed the same broad peaks at the corresponding elution times for Trn α and β. Given that the chromatogram for the free peptide in the supernatant of the Trn αβ liposome suspension did not show any peaks and, considering that peaks for the two peptides could be detected at concentrations as low as 2.5 μg/mL, the encapsulation efficiency can be hypothesised to be higher than 97.5 %.

It was attempted to quantify the concentration of thuricin CD released from αβ liposomes into FaSSGF and FaSSIF by RP-HPLC. As was previously stated, peaks for peptides α and β could be detected at concentrations as low as 2.5 μg/mL with the RP-HPLC method used. None of the release media FaSSGF or FaSSIF at any time points showed peaks for Trn α nor β, indicating that thuricin CD is not released from the αβ liposomes, probably due to their low solubility in the release media. This behaviour is commonly seen for hydrophobic drug release analysis using standard protocols (D’Addio et al., 2016; Leganés, 2022).

**Fig. 7:**
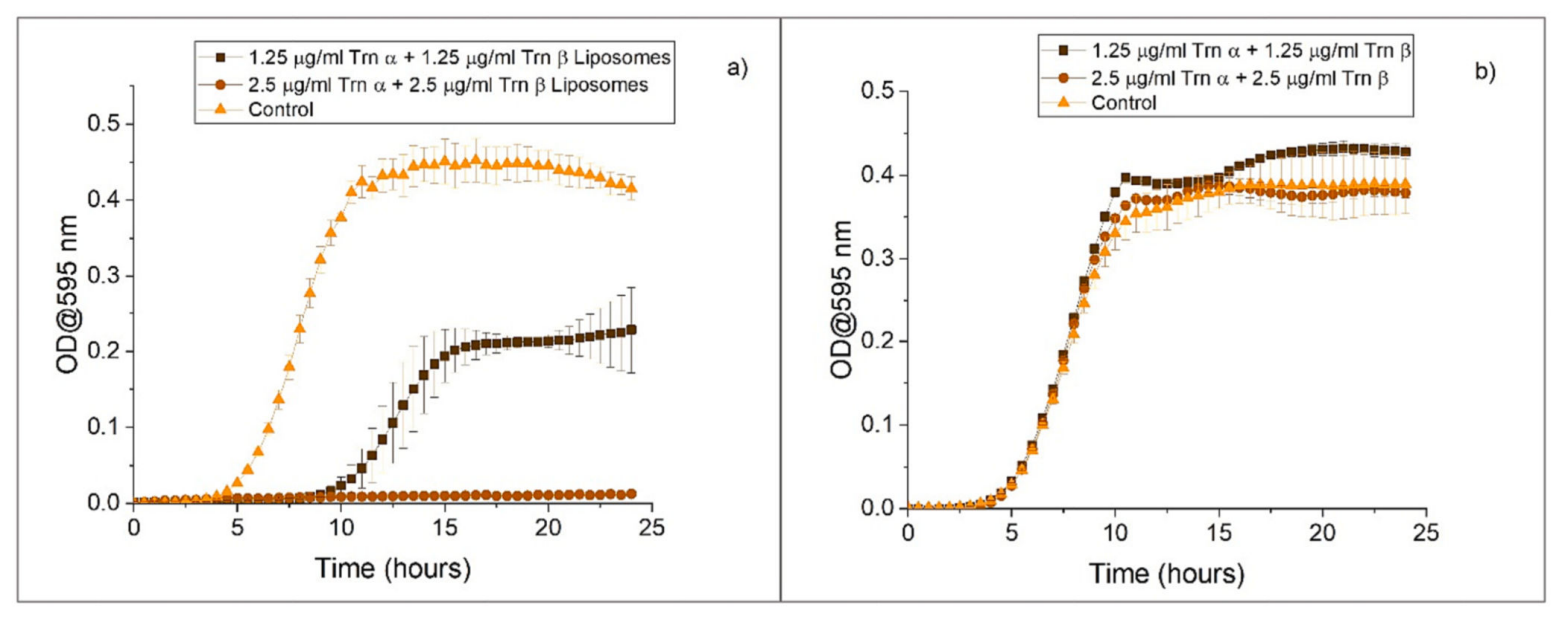
Activity of a) Trn αβ liposomes with a control of blank liposomes and b) Lyophilised Trn α and β suspended in acetate buffer with a control of just acetate buffer against *L. monocytogenes*.

The activity of the Trn αβ liposome suspension against *L. monocytogenes* was analysed and compared to lyophilized Trn α and β, which were suspended in acetate buffer as this is the same buffer present in the liposome suspension. As shown in Fig. 7a, the loaded liposomes demonstrate complete killing at a concentration of 2.5 μg/mL Trn α +2.5 μg/mL Trn β. At half of this concentration bacterial growth was inhibited by approximately 50 %. Blank liposomes were set as the control and normal bacterial growth can be observed, indicating that the liposomes themselves do not have activity against *L. monocytogenes*. These results can be compared to the results obtained with thuricin CD previously dissolved in 70 % IPA 0.1 % TFA in water in the first section, suggesting that liposomes are good vehicle for thuricin CD. On the other hand, in Fig. 7b, the peptides Trn α and β directly suspended in acetate buffer do not demonstrate any activity at the same concentrations.

**Fig. 8:**
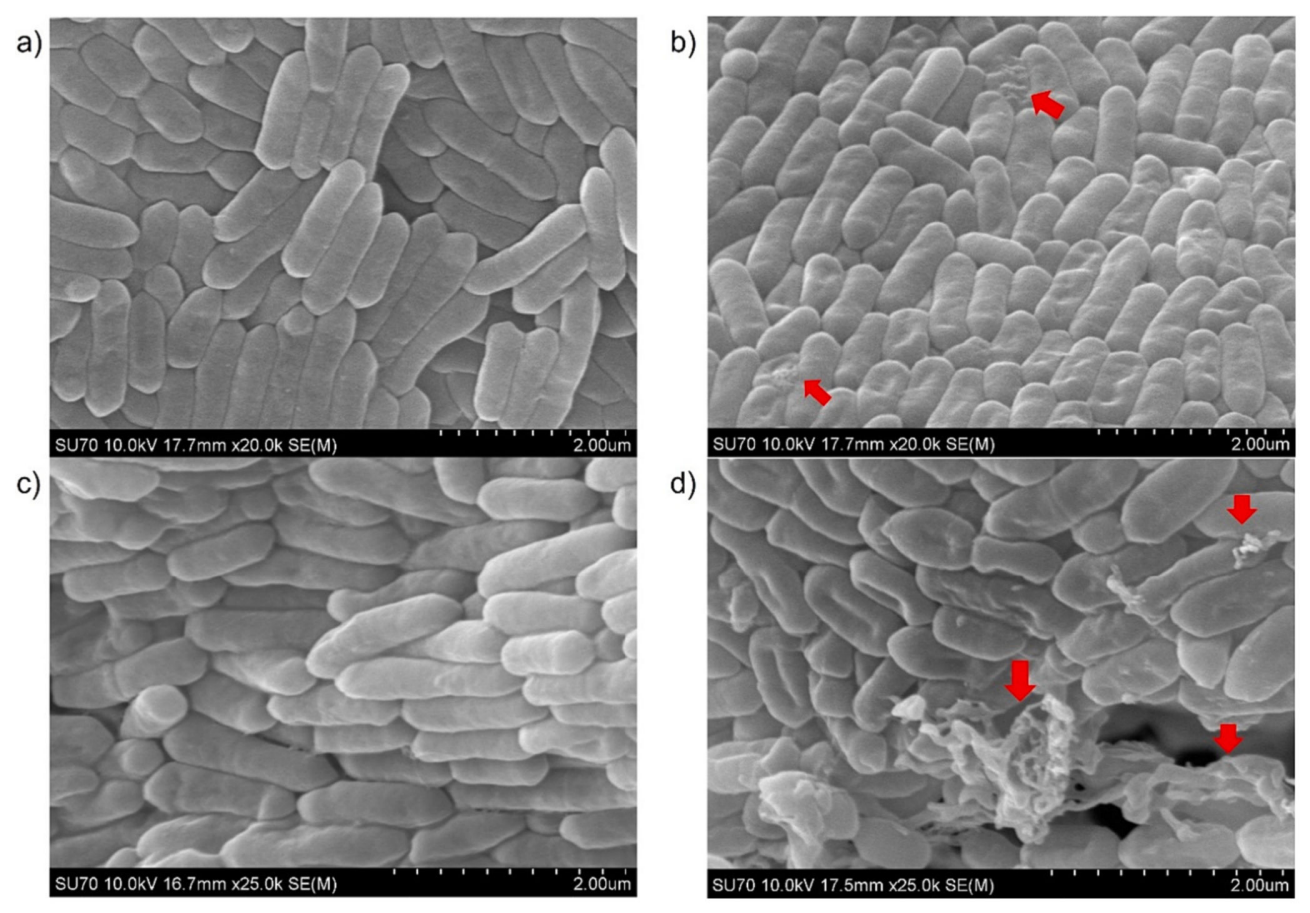
SEM images of *L. monocytogenes* cells after exposure for 24 h to a) PBS, b) 2.8 μg/mL Trn α + 2.8 μg/mL Trn β previously dissolved in 70 % IPA 0.1 % TFA in water c) blank liposome suspension d) liposome suspension loaded with 2.8 μg/mL Trn α + 2.8 μg/mL Trn β. Red arrows indicate punctures and leakage of intracellular contents seen on the bacterial cells. (For interpretation of the references to colour in this figure legend, the reader is referred to the web version of this article.)

Bacterial cells treated with just PBS displayed the typical rod-shaped morphology of *L. monocytogenes* (Fig. 8a). *L. monocytogenes* cells after treatment with 2.8 μg/mL Trn α + 2.8 μg/mL Trn β previously dissolved in 70 % IPA 0.1 % TFA in water, and diluted in PBS, revealed punctures and changes in morphology in some cells (Fig. 8b). Fig. 8c shows *L. monocytogenes* treated with blank liposomes suspension in acetate buffer and the cells have the typical expected morphology, similar to the PBS control. In contrast, Fig. 8d demonstrates that Trn αβ loaded liposome suspension, at the same added concentration of dissolved thuricin CD, caused significant morphological changes, punctures, and leakage of cell contents not seen in the other samples, suggesting a more potent antimicrobial effect. Moreover, the bacterium pellet size after treatment was bigger for the controls than for dissolved thuricin CD and Trn αβ liposomes, confirming this higher antimicrobial activity. Previous studies have proposed that drug-loaded liposomes have superior anti-microbial activity compared to free drugs because they are structurally similar to bacterial membranes, facilitating greater interaction and fusion, and resulting in improved antibiotic delivery (Ferreira, 2021).

The particle size distribution and PDI of the αβ liposome suspension after exposure to FaSSGF with pepsin and FaSSIF with pancreatin were measured. Incubation times of 15 and 30 min for FaSSGF were chosen to mimic the time for complete emptying of fasting stomach. For FaSSIF, 3 h was chosen as the incubation time to mimic the transit time through the entire small intestine in fasted state (Mudie et al., 2010).

**Fig. 9:**
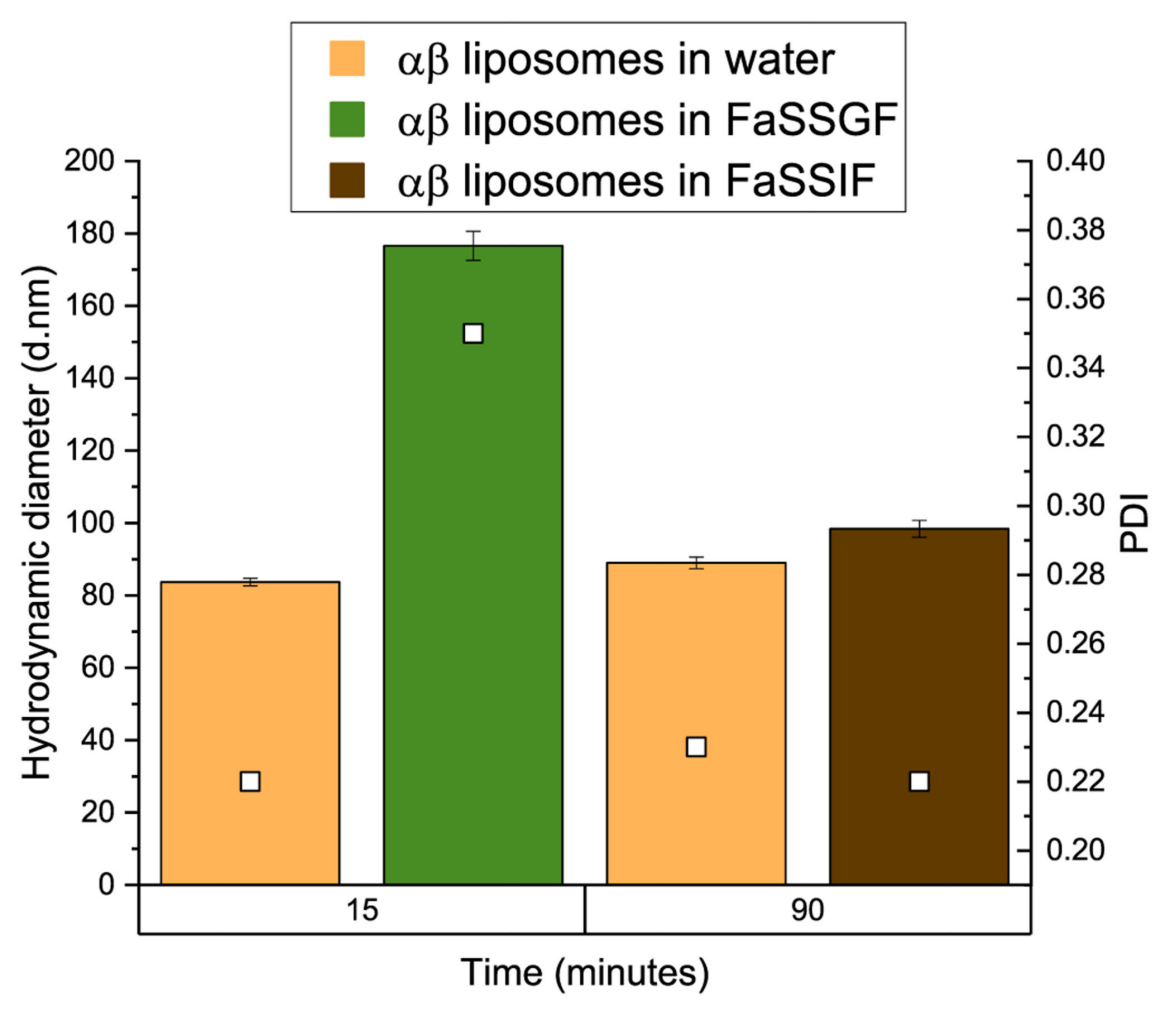
Stability of Trn αβ liposome suspension in FaSSGF and FaSSIF with enzymes (pepsin and pancreatin respectively) measured by size (coloured bars) and PDI (white squares).

It can be seen from Fig. 9 that Trn αβ liposome suspension increased in size and PDI by almost double in FaSSGF in 15 min compared to their size in water. Results after 30 min are not shown because the value obtained exceeded the limits of detection of the measurement equipment. The appearance of this solution was turbid compared to the liposome’s suspension. The acidic FaSSGF could be affecting the surface charge of the liposomes and therefore, promoting aggregation, which is reflected by the increase in size and polydispersity index. Previous studies have also shown an increase in liposome size after exposure to gastric fluid (Lee et al., 2005).

Trn αβ liposomes exposed to FaSSIF maintained their size and PDI (Fig. 9). Trn αβ liposomes suspension in FaSSIF did not show any visual difference from the liposomes in water. It has been stated previously in literature, that liposomes are commonly affected by bile salts and pancreatin, resulting in structural instability and consequent leakage (He et al., 2019). However, it has also been stated that, one of the strategies to improve the stability of liposomes for oral delivery, is to optimize the lipid composition (He et al., 2019). Some of the most common strategies is to include cholesterol in the formulations or lipids with higher transition temperatures as this will generate more rigid liposomes (Kokkona et al., 2000). Here, HSPC and DPPG Na are used in the Trn αβ liposome formulation, two lipids with transition temperatures of 53 and 41 °C respectively as stated by the manufacturer. This could be contributing to the stability of the liposomes which is reflected by their stability after over 3 h of exposure to FaSSIF. An additional factor that might be contributing to liposome stability is its negative charge, which would repel the negatively charged bile salts. Nevertheless, there is very little information available about negatively charged liposomes for oral delivery (Kisel, 2001).

**Fig. 10:**
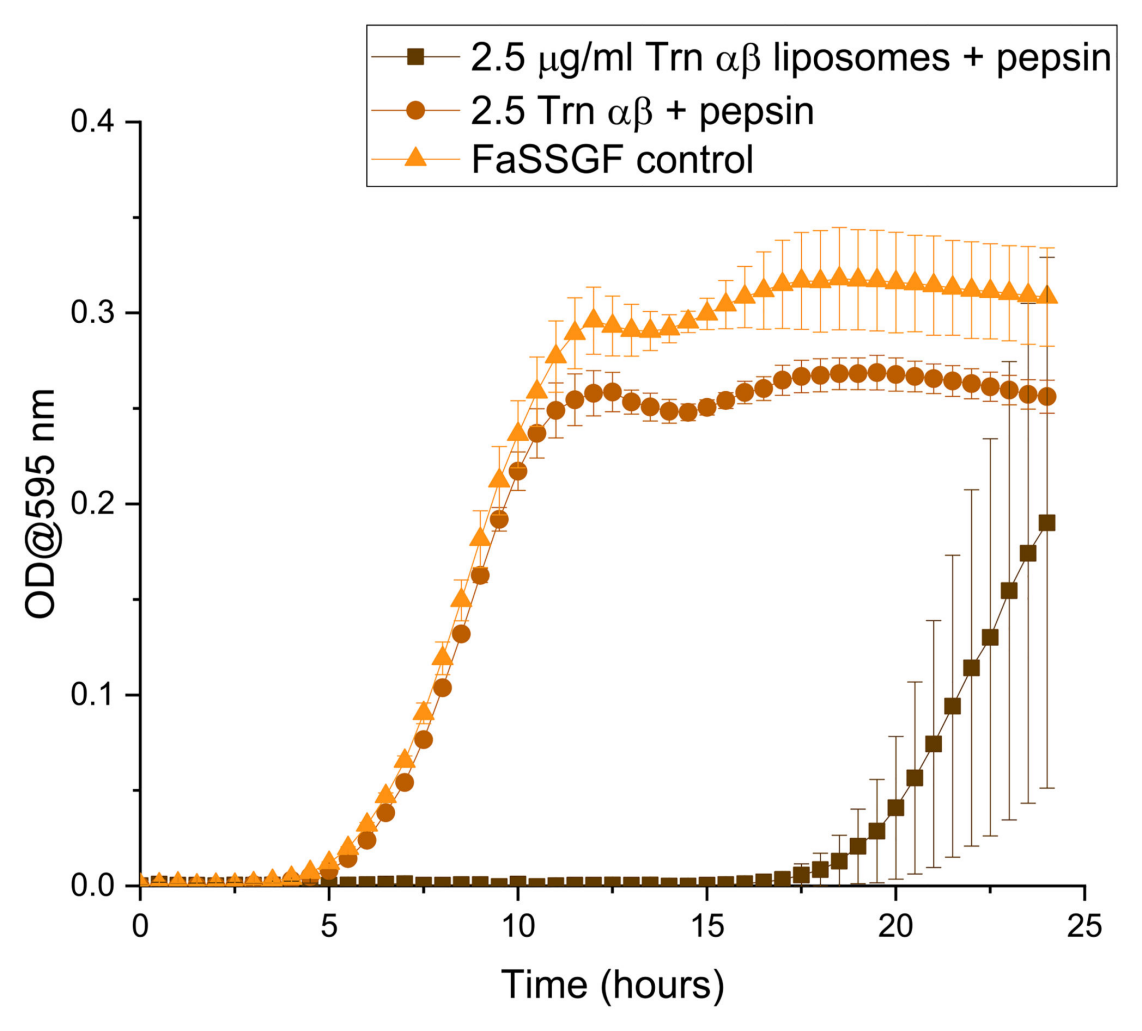
Activity of thuricin CD and Trn αβ loaded liposomes against *L. monocytogenes* ATCC 1916 after exposure to pepsin in FaSSGF.

The activity of the Trn αβ liposome suspension after exposure to pepsin in FaSSGF was evaluated to determine whether liposomes could protect the peptide from degradation by this enzyme in the gastric tract (Fig. 10). Liposomes loaded with Trn α and β displayed complete inhibition of bacterial growth for up to 17.5 h after exposure to pepsin in contrast with the same concentration of dissolved Trn α and β that completely lost activity after being exposed to pepsin in FaSSGF for 30 min, with the growth curve resembling that of the control containing only pepsin in FaSSGF and *L. monocytogenes*. These findings support previous reports that liposomes can protect peptides or proteins from enzymatic degradation, as demonstrated by the maintenance of thuricin CD activity against *L. monocytogenes* after exposure to pepsin, despite the observed particle size increase(Uhumwangho and Okor, 2009). This could mean that even though particles aggregated, the vesicle membranes maintained their integrity and prevented pepsin from degrading the peptides.

**Fig. 11:**
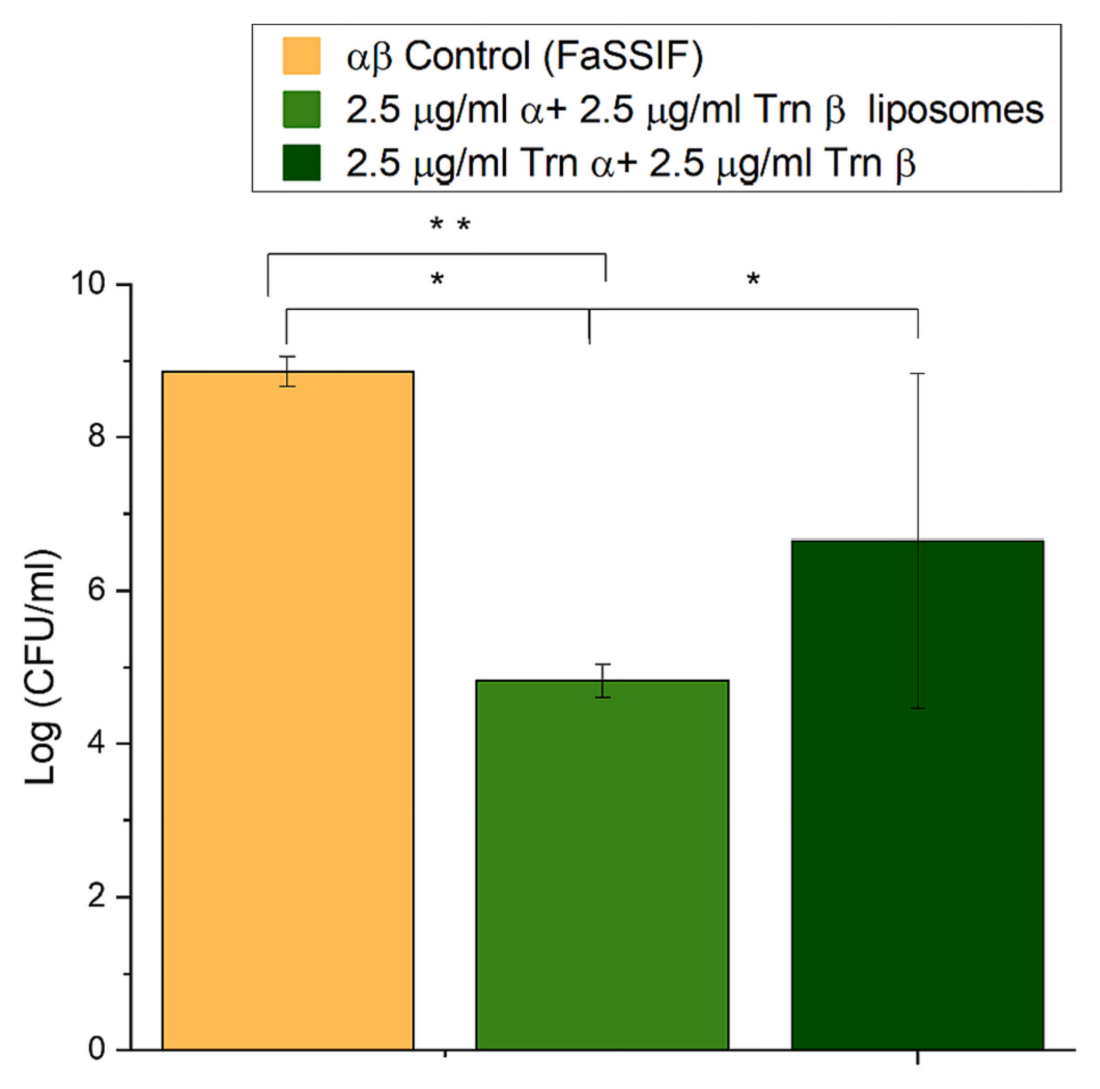
Activity of dissolved thuricin CD and Trn αβ loaded liposomes against *L. monocytogenes* ATCC 1916 displayed as CFU/mL after exposure to FaSSIF. * not significantly different ** significantly different p < 0.05.

The activity of Trn αβ liposome suspension after exposure to FaSSIF after 3 h was also evaluated by total plate counts (Fig. 11). A 4.04 ± 0.17 log reduction or 99.99 % inhibition of bacterial growth was obtained with a concentration of 2.5 μg/mL of each peptide α and β encapsulated in the liposome’s suspension compared to the control sample (acetate buffer with FaSSIF at the same proportions as the samples). Both samples were significantly different when evaluated statistically. A concentration of 2.5 μg/mL of the α and β peptides previously dissolved in 70 % IPA 0.1 % TFA in water showed a 2.21 ± 2.30 log reduction or 91.06 %, which is lower than the inhibition obtained by Trn αβ liposomes. However, dissolved thuricin CD peptides showed no significant difference in activity from the Trn αβ liposomes nor the control in the statistical analysis due to the large standard deviation.

**Fig. 12:**
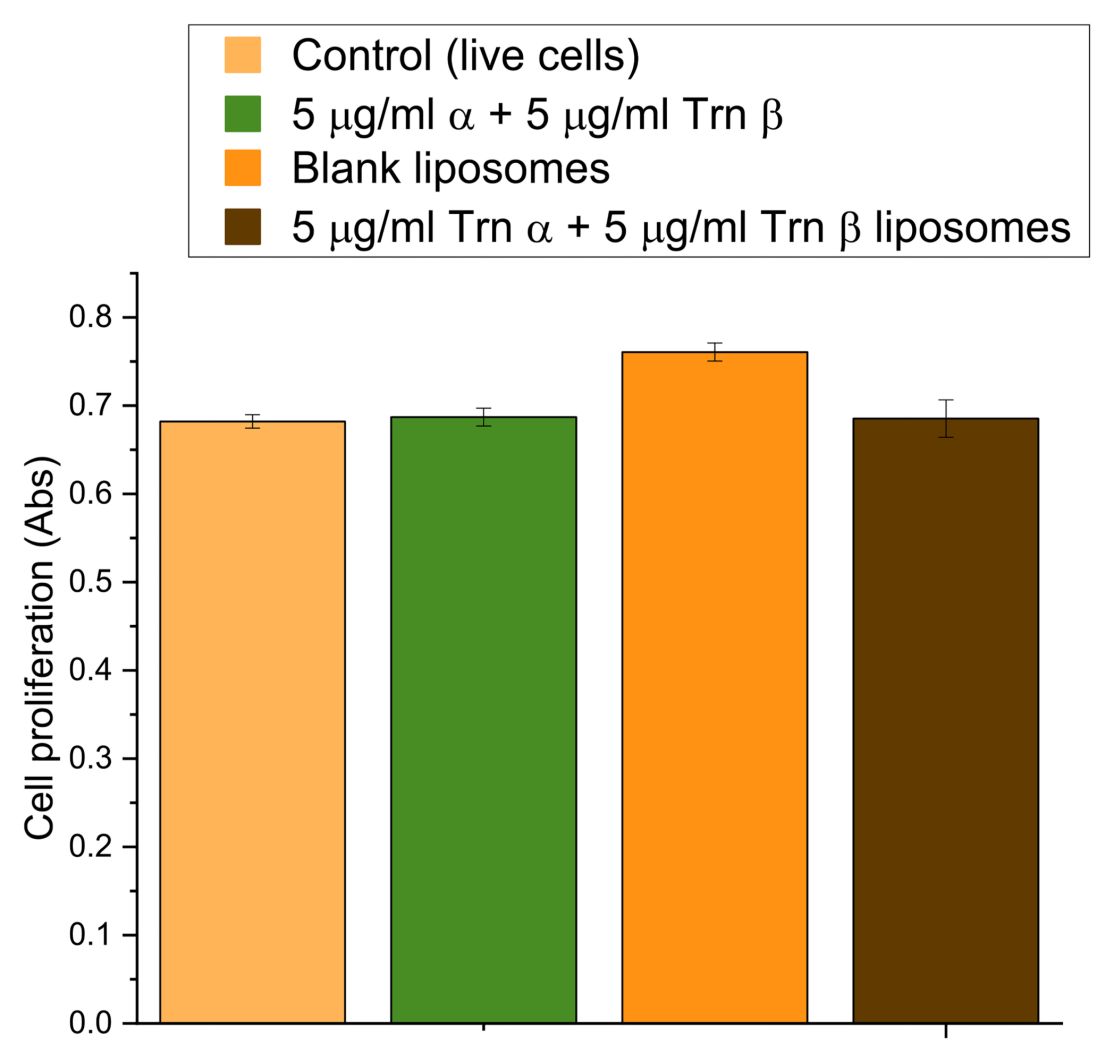
Biocompatibility of thuricin CD (5 μg/mL Trn α + 5 μg/mL Trn β), thuricin CD loaded liposomes (5 μg/mL Trn α + 5 μg/mL Trn β), and blank liposomes against Caco-2 cells displayed by absorbance after 24 h incubation.

The biocompatibility of thuricin CD to mammalian cells has not been previously reported. In this study, the results of a Presto blue cytotoxicity assay of dissolved thuricin CD and the Trn αβ liposome suspension with epithelial intestinal cells are presented. After incubation for 24 h, none of the samples exhibited any significant difference in absorbance compared to the control group of living cells (as shown in Fig. 12). These findings suggest that neither thuricin CD nor Trn αβ liposomes pose any harm to Caco-2 cells and may be safe for oral delivery.

## 4 Conclusion

Dual acting antimicrobial peptides of thuricin CD were shown to be poorly soluble and unstable in simulated gastrointestinal fluids containing enzymes, leading to a loss of activity over time. However when the peptides were encapsulated into anionic liposomes (Trn αβ liposomes), they exhibited the same activity as free thuricin CD when an organic solvent is used for dissolution of the hydrophobic peptides, maintained stability and activity in FaSSGF (with pepsin) and FaSSIF and did not show toxicity to intestinal epithelial cells. The thuricin CD-loaded liposomes are stable for up to 21 days at room temperature and 60 days at 4 °C, making them a promising vehicle for the oral delivery of the antimicrobial bacteriocin, thuricin CD.

## Supplementary Material

Appendix A. Supplementary data

## Figures and Tables

**Table 1:** Composition of FaSSIF and FaSSGF.

Media	pH	Taurocholate	Phospholipids	Sodium	Chloride	Phosphate
FaSSIF	6.5	3 mM	0.75 mM	148 mM	106 mM	29 mM
FaSSGF	1.6	0.08 mM	0.02 mM	34 mM	59 mM	—

## Data Availability

Data will be made available on request.
