# Supplementary material for: Anionic liposome formulation for oral delivery of thuricin CD, a potential antimicrobial peptide therapeutic
Source: Int J Pharm. Author manuscript; Available in PMC 2024 Apr 1. (PMC7615751; doi:10.1016/j.ijpharm.2024.123918)
Supplement: Appendix A. Supplementary data [file EMS194523-supplement-Appendix_A__Supplementary_data.docx]

**Anionic liposome formulation for oral delivery of thuricin CD, a potential antimicrobial peptide therapeutic**

**Camila Viera Herrera^a^, Paula M. O’Connor^b,c^, Poonam Ratrey^a^, R Paul Ross^c,d^, Colin Hill^c,d^, Sarah P. Hudson^a,^***

^a^ Department of Chemical Sciences, Bernal Institute, University of Limerick, Ireland,

^b^ Food Biosciences , Teagasc, Moorepark Food Research Centre, Fermoy, Co. Cork, Ireland,

^c^ APC Microbiome Ireland Cork, Cork, Ireland.

^d^ School of Microbiology, University College Cork, College Road, Cork, Ireland.

*Email: sarah.hudson@ul.ie

**Supplementary information**

**Section A:**

**Production and purification of Thuricin CD**

Trn α and Trn β peptides were produced and purified with a varying yield of 2 to 5 mg/L. Structures of each peptide are shown in Figure S1.


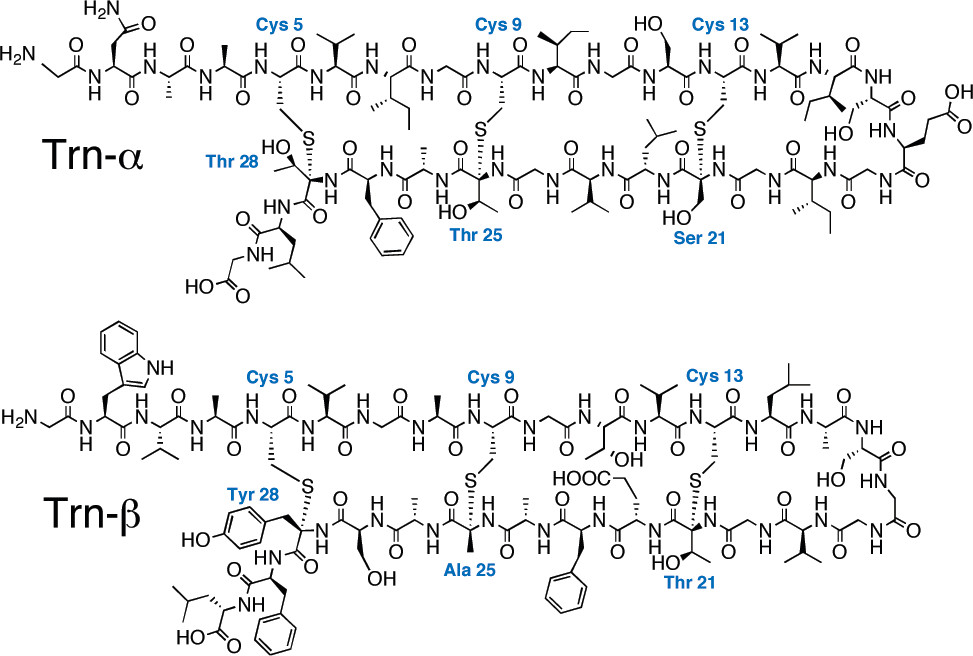


Figure S1. Chemical structures of Trn α and Trn β [1].

**Section B:MALDI-TOF mass spectrometry**

Mass spectrometry was performed on the purified thuricin CD to confirm its production. The individual peptides were resuspended from powder in 70% IPA 0.1% TFA in water at a concentration of 1 mg/mL. Sinapinic acid (SA) was used as the matrix. 0.7 µl of 4 mg/ml sinapinic acid solution in a 1:1 (vol) acetonitrile: methanol solution was added to the plate and left to evaporate. Then, a 10 mg/mL SA solution in 60% ACN 0.1% TFA in water was prepared. This was mixed in a 1:1 volume ratio with each peptide solution. 0.3 µL of the mix were added to the plate and left to evaporate. The same protocol was followed for the calibrant used which was “Peptide II”. The plate was loaded into MALDI-TOF Ultraflex from Bruker and RP-700-3500 kDa mode was selected. Shoot intensity was approximately 70 %.


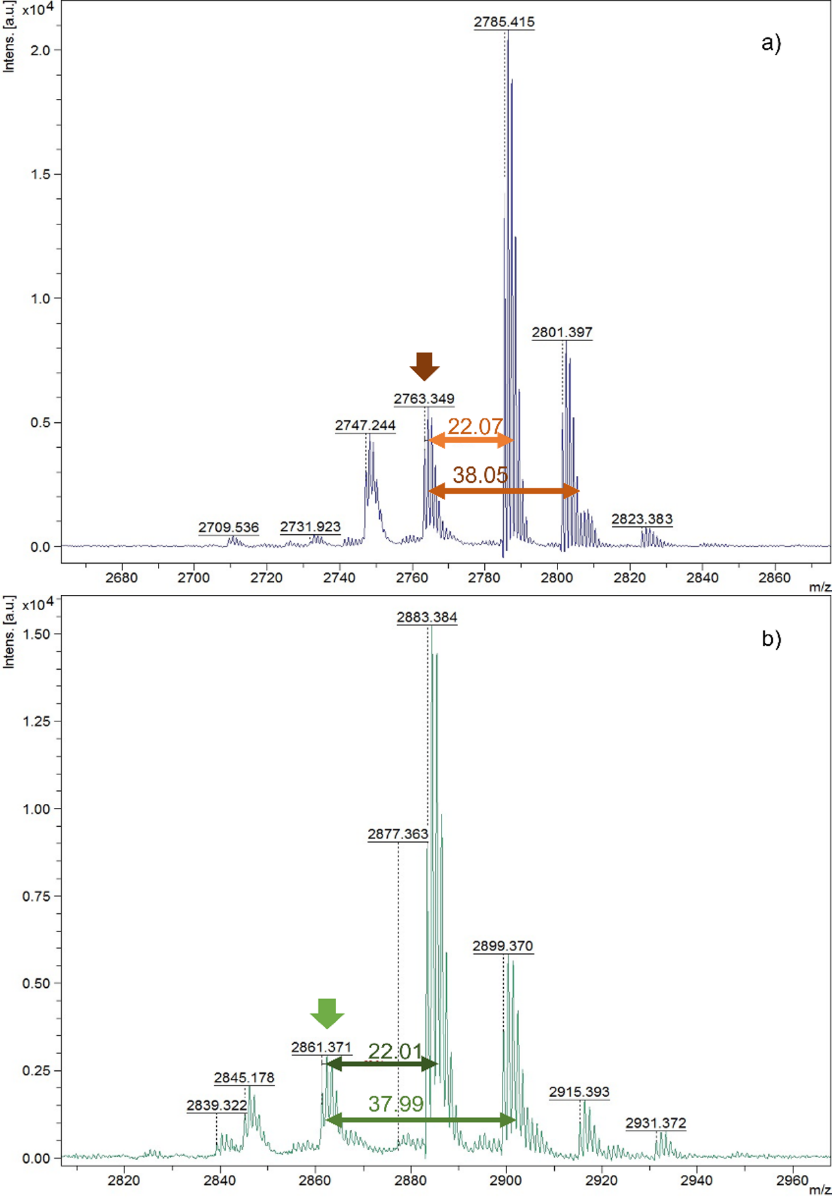


Figure S2. Mass spectrum of the products obtained from the production and purification method corresponding to a) Trn α b) Trn β.

Peaks corresponding to masses of 2763.35 Da and 2861.37 Da were detected for each peptide marked by the green and brown arrows in Figure S2. In both observed peaks, a prominent peak 22 mass units to the right can also be observed corresponding to the [M+Na] + adduct. 38 mass units to the right, another peak which would correspond to [M+K] + adduct is also observed. These are commonly found when using positive reflectron mode in mass spectrometry for biological samples [2].

**Section C:**

**Activity of dissolved thuricin CD after exposed to FaSSGF**


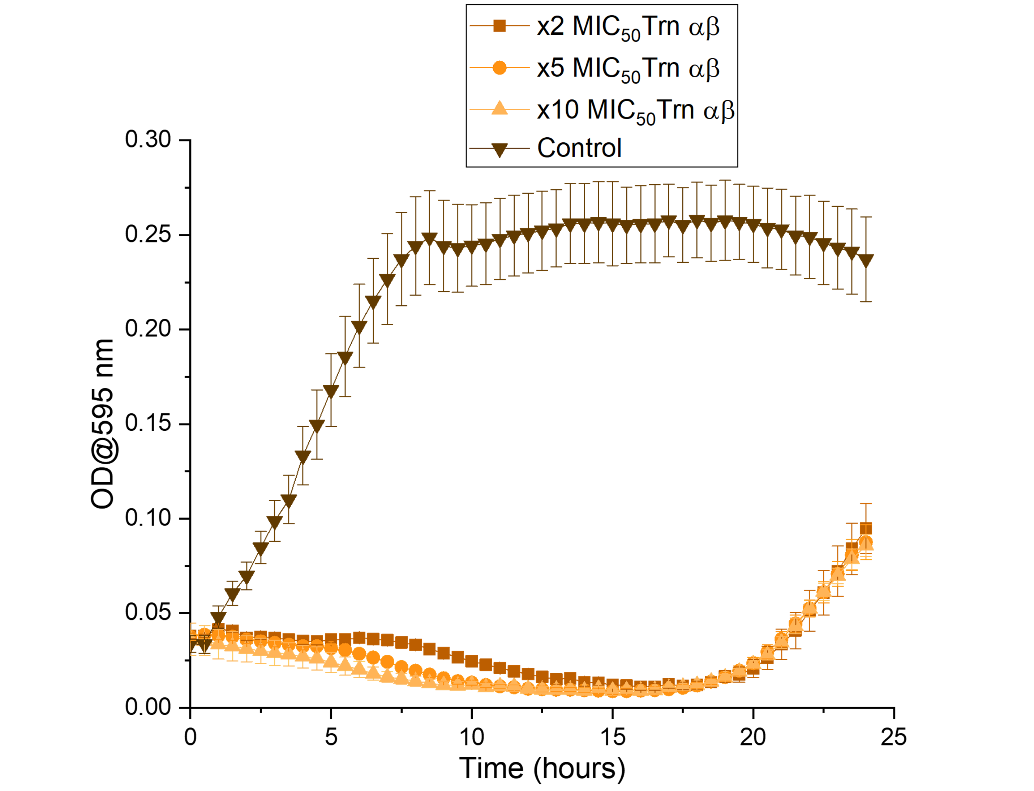


Figure S3. Biological activity of thuricin CD lyophilised peptides pre-dissolved in 70% IPA 0.1% TFA in water and diluted in FaSSGF at concentrations of 2, 5, and 10 times the MIC_50_ against *L. monocytogenes* at a 1:1 molar ratio*.*

**References**

[1] C. S. Sit, R. T. McKay, C. Hill, R. P. Ross, and J. C. Vederas, “The 3D structure of Thuricin CD, a two-component bacteriocin with cysteine sulfur to α-carbon cross-links,” *J Am Chem Soc*, vol. 133, no. 20, 2011, doi: 10.1021/ja201802f.

[2] M. Janda, B. K. B. Seah, D. Jakob, J. Beckmann, B. Geier, and M. Liebeke, “Determination of Abundant Metabolite Matrix Adducts Illuminates the Dark Metabolome of MALDI-Mass Spectrometry Imaging Datasets,” *Anal Chem*, vol. 93, no. 24, pp. 8399–8407, Jun. 2021, doi: 10.1021/acs.analchem.0c04720.
